# High-throughput fluorescent-based NKCC functional assay in adherent epithelial cells

**DOI:** 10.1186/1471-2121-14-16

**Published:** 2013-03-18

**Authors:** Monica Carmosino, Federica Rizzo, Silvia Torretta, Giuseppe Procino, Maria Svelto

**Affiliations:** 1 Department of Biosciences, Biotechnologies and Biopharmaceutics, University of Bari, Via Amendola 165/A, Bari, 70126, Italy; 2 Department of Sciences, University of Basilicata, Via dell’Ateneo Lucano, Potenza, 85100, Italy

**Keywords:** NKCC activity, Fluorescent assay, High-throughput, Drug screening

## Abstract

**Background:**

The kidney-specific NKCC cotransporter isoform NKCC2 is involved in the Na^+^ reabsorption in the Thich Ascending Limb (TAL) cells and in the regulation of body fluid volume. In contrast, the isoform NKCC1 represents the major pathway for Cl^-^ entry in endothelial cells, playing a crucial role in cell volume regulation and vascular tone. Importantly, both NKCC isoforms are involved in the regulation of blood pressure and represent important potential drug targets for the treatment of hypertension.

**Results:**

Taking advantage of an existing Thallium (Tl^+^)-based kit, we set up a Tl^+^ influx-based fluorescent assay, that can accurately and rapidly measure NKCC transporter activity in adherent epithelial cells using the high-throughput Flex station device. We assessed the feasibility of this assay in the renal epithelial LLC-PK1 cells stably transfected with a previously characterized chimeric NKCC2 construct (c-NKCC2). We demonstrated that the assay is highly reproducible, offers high temporal resolution of NKCC-mediated ion flux profiles and, importantly, being a continuous assay, it offers improved sensitivity over previous endpoint NKCC functional assays.

**Conclusions:**

So far the screening of NKCC transporters activity has been done by ^86^Rb^+^ influx assays. Indeed, a fluorescence-based high-throughput screening method for testing NKCC inhibitors would be extremely useful in the development and characterization of new anti-hypertensive drugs.

## Background

The Na-K-2Cl cotransporters, NKCC1 and NKCC2, are members of the superfamily of electroneutral cation-coupled co-transporters (*SLC12A*), playing crucial roles in the cellular and body fluid homeostasis.

Located in the apical membrane and subapical vesicles in the thick ascending limb of the Henle’s loop in the mammalian kidney, NKCC2 is responsible for reabsorbing about 20% of filtered NaCl. In the *macula densa* it is also essential for tubuloglomerular feedback, the cross talk mechanism that finely tunes tubular reabsorption in response to variations of the glomerular filtration rate. Indeed, NKCC2 plays key roles in regulating body salt levels and blood pressure [1,2]. NKCC2 is the pharmacological site of action for loop diuretics; defects in its operation cause Bartter’s disease while its upregulation may contribute to the onset of essential hypertension.

Despite its importance, relatively little work has been carried out on NKCC2, mainly due to difficulties in expressing NKCC2 in a functionally-competent form in epithelial cells [3,4]. Indeed, chimeric [5,6] or tagged [7] recombinant proteins have been functionally expressed in mammalian cells and in *Xenopus* oocytes. These studies provided important information about transport kinetics and ion affinities [8,9] displayed by different NKCC2 constructs.

Most of the information on the activity of NKCC2 is deduced from that of NKCC1 due to the high homology on the behavior of this closely related isoform, which has been successfully expressed in cultured cells and extensively studied.

NKCC1 represents the major pathway for Cl^-^ entry in mammalian cells, playing a crucial role in cell volume regulation [10]. NKCC1 is also involved in the pathological secretion of cystic fluid in the Autosomal Polycystic Kidney disease [11] and modulator of vascular tone [12].

The functional studies of both NKCC1, NKCC2 and NKCC1/NKCC2 chimeric constructs have been performed so far using the ^86^Rb^+^ assay [13-15] or, alternatively, the NKCC-mediated NH4^+^ uptake assay measured with a pH-sensitive fluorescent dye [16].

Rb^+^ is the closest-related potassium analog and its isotope (86) is characterized by the emission of high-energy β and γ radiations, which allow its quantification by Cerenkov counting without the need of liquid scintillation fluid addition.

However, the principal drawback of ^86^Rb^+^ lies in the potential toxicity and health hazard associated with radioactivity. As a consequence, many labs are reluctant to use the ^86^Rb^+^-based radioactive flux assay format for the analysis of NKCC activity.

Alternatively, ^86^Rb^+^ isotope can be substituted with non-radioactive ^85^Rb^+^ and its amount quantified by atomic absorption spectroscopy [17]. However, both assays suffer from poor temporal resolution.

In this report, we describe the development of a fluorescent-based influx assay that can accurately and rapidly measure the activity of a chimeric NKCC2 construct expressed at the apical membrane of polarized epithelial cells.

In agreement with previous workers [5,6] we found that the presence of the N-terminus of NKCC2 in any construct appears to prevent functional expression in HEK-293 cells as well as stable expression in epithelial cells such as MDCK and LLC-PK1 cells.

Indeed, we circumvent this problem by the use of a chimeric NKCC1-NKCC2 construct, which shares crucial features of either NKCC1 or NKCC2 isoforms, such as the high predisposition to the stable expression in epithelial cells and the selective localization at the apical membrane, respectively.

Our functional assay is based on the use of Thallium (Tl^+^), instead of Rb^+^, as the K^+^ tracer. This is possible because of the selective permeability of all K^+^ ion channels and transporters for Tl^+^ and the strong driving force for Tl^+^ entry into the cells when the channels-transporters are activated [17-19].

We took advantage of the availability of a Tl^+^-sensitive, fluorescence-based ion flux indicator successfully used in a high-throughput assay as previously reported [20]. Tl^+^ binds with high affinity to the corresponding K^+^ ion site on c-NKCC2 and once transported within the cytoplasm, where it is naturally absent, it associates with the halide-sensitive fluorescent dye, causing a fluorescence increase that can be detected by the Flex station Device. The most important advantages of this method are 1- the high temporal resolution compared to the end point assays, 2- a more direct measurement of the NKCC transport activity compared to the indirect assays.

## Results

### Characterization of the cellular model

We sought to assess the feasibility of this assay in the renal epithelial LLC-PK1 cells stably transfected with a previously characterized chimeric NKCC2 construct (c-NKCC2) [6,21,22]. This cell line was tested for the polarized expression of the Na-K-ATPase and c-NKCC2 by confocal immunofluorescence when grown on coverslip, a condition that mimics that of cells grown in the 96 well microplates. C-NKCC2 was detected using an antibody against the HA tag, expressed at the N-terminal tail of the protein [6], thus specifically identifying the transfected c-NKCC2 isoform.

**Figure 1:**
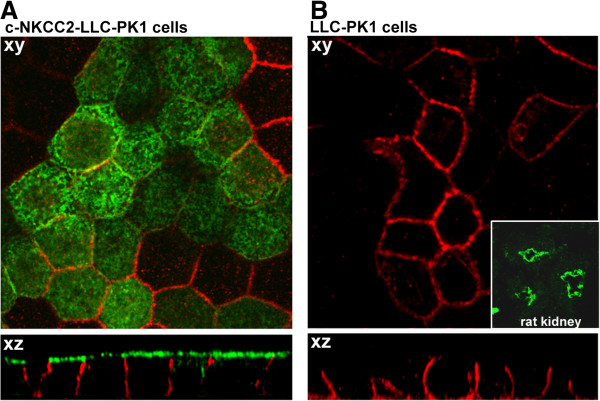
**A) Immunofluorescence confocal analysis of Na-K-ATPase (red signal) and c-NKCC2 (green signal) in c-NKCC2-transfected LLC-PK1 cells grown on coverslips.** The merged signal of planar xy and xz projections was depicted. Na-K-ATPase and c-NKCC2 are selectively expressed at the basolateral and apical membrane respectively. **B**) Immunofluorescence confocal analysis of Na-K-ATPase (red signal) and the endogenous NKCC2, (green signal) in untransfected LLC-PK1 cells grown on coverslips. The merged signal of planar xy and xz projections was depicted. Na-K-ATPase was selectively expressed at the basolateral membrane whereas the endogenous NKCC2 was absent. *Inset*: Immunolocalization of the endogenous NKCC2 in a rat kidney section.

As shown in Figure 1A c-NKCC2 (green labeling) and Na-K-ATPase (red labeling) are selectively expressed at the apical and basolateral membrane of LLC-PK1 respectively, demonstrating that in the condition in which cells were assayed for NKCC2 functional activity, LLC-PK1 cells are correctly polarized. Moreover, untransfected LLC-PK1 cells were checked for the endogenous expression of NKCC2 using a specific anti-NKCC2 antibody. As show in Figure 1B, although the expression of the Na-K-ATPase was again clearly appreciable (red labeling), the endogenous expression of NKCC2 was undetectable in any cellular compartment.

Of note, the same antibody well recognized the endogenous NKCC2 in rat kidney sections (Figure 1B, inset). In conclusion, the c-NKCC2-transfected LLC-PK1 cells will allows us to follow only the activity of the ectopically expressed c-NKCC2 protein.

### Tl^+^ influx assay in mock and c-NKCC2 transfected LLC-PK1 cells

**Figure 2:**
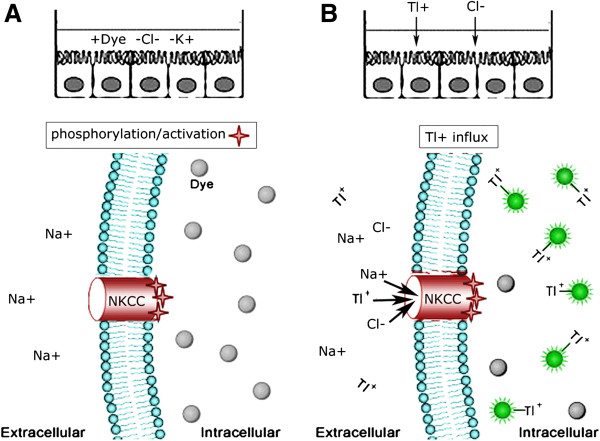
**Scheme of the experimental design modified from **http://probes.invitrogen.com/media/pis/mp10016.pdf**. A**) Cells cultured in the 96 well plate were preincubated with the Tl^+^-sensitive FluxOR dye (Dye) in a Cl^-^-free loading buffer (−Cl^-^) to promote c-NKCC cotransporter activation. The loading buffer was also K^+^-free (−K^+^) to rule out the competition of K^+^ with Tl^+^ at the binding site onto the c-NKCC cotransporter. FluxOR, loaded within the cytoplasm, is quenched in the absence of Tl^+^ ions. **B**) The assay is started with the following addition of Tl^+^ and Cl^-^ in the assay plate. Tl^+^ flows inside the cells along with Na^+^ and Cl^-^ through the c-NKCC cotransporter. Upon binding to cytosolic Tl^+^, the FluxOR dye exhibits a strong increase in fluorescence intensity.

Mock- and c-NKCC2-transfected LLC-PK1 cells were incubated in chloride- and potassium-free assay buffer in the presence of the FluxOR™ dye to simultaneously achieve both activation of the c-NKCC2 cotransporter and cell loading with the dye (Figure 2A). The absence of K^+^ from the assay buffer was necessary to avoid the competition with Tl^+^ on c-NKCC2. Then we analyzed the Tl^+^ and Cl^-^ influx in these cells by monitoring the effect of successive addition of Tl^+^ and Cl^-^ on the fluorescence signal dynamics (Figure 2B) as recorded by the Flexa microplate reader.

**Figure 3:**
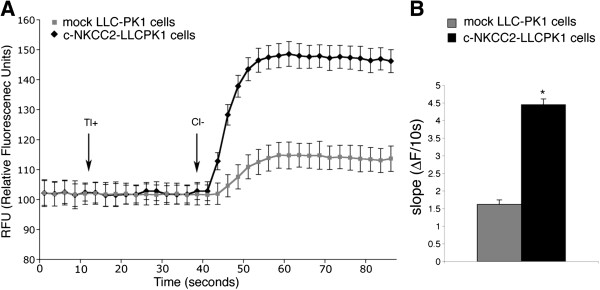
**Tl^+^ influx assay in LLC-PK1 cells. A**) Mean signal traces (average from 6 wells) obtained with the fluorometric imaging plate reader (FlexStation). LLC-PK1 cells either mock-transfected or stable transfected with c-NKCC2 construct were shown. Tl^+^ influx was initiated by Cl^-^ addition. A robust Cl^-^-dependent Tl^+^ influx was observed in c-NKCC2-transfected but not in mock-transfected LLC-PK1 cells. **B**) The initial rate of Tl^+^ influx registered during the assay shown in A was reported. The rate of Tl^+^ influx was increased by about 3-fold upon c-NKCC2 expression in LLC-PK1 cells. Values are means ± SE of 3 independent experiments. Student’s t test for unpaired data, *p < 0.0001.

The kinetics of the fluorescence signal was recorded over a period of 90 s (Figure 3A). In the absence of Cl^-^ in the assay buffer, Tl^+^ addition did not induce any increase in fluorescence suggesting that, in this experimental condition, Tl^+^ was not transported within the cells through any other K^+^ transporter expressed at the apical side of the monolayer. Moreover, this result confirms that, in this experimental condition, LLC-PK1 cells form a tight epithelium that makes the basolateral Na^+^-K^+^-ATPase inaccessible to the Tl^+^ ions added at the apical side.

Interestingly, a robust Tl^+^ influx was observed only after Cl^-^ addition in c-NKCC2- expressing LLC-PK1 cells but not in mock-transfected LLC-PK1 cells in which only a weak increase in fluorescence was observed (Figure 3A, mock and c-NKCC2-LLC-PK1 cells). Indeed, as predicted by the ectopic expression of c-NKCC cotransporter, Tl^+^ transport within the cell required the presence of external Cl^-^.

The c-NKCC2-driven fluorescence signal recorded after Cl^-^ addition displays a rapid linear increased phase within the first 10-15 s, followed by a slower increase and a *plateau* phase. In our experiments kinetic measurement of the transporter activity followed the same fundamental concepts and principles as measurement of enzyme activity. Thus, c-NKCC2 activity was most accurately represented by the initial rate of Tl^+^ transport, which can be calculated using the initial linear phase of the curve. The initial rate of Cl^-^-dependent Tl^+^ influx observed in c-NKCC2 transfected cells is about 3-fold over the background signal registered in mock-transfected LLC-PK1 cells (Figure 3B).

To further confirm that the observed Tl^+^ influx was actually mediated by c-NKCC2, we assessed the effects of the known inhibitor of NKCC cotransporters, Furosemide.

**Figure 4:**
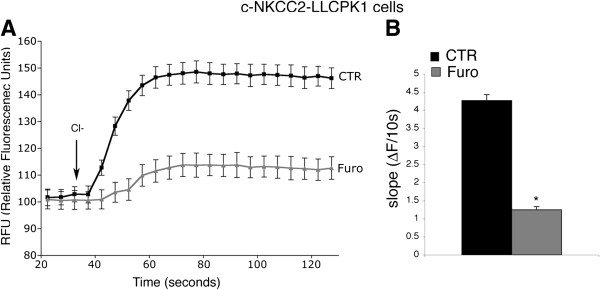
**Tl^+^ influx assay for c-NKCC2 activity in c-NKCC2-expressing LLC-PK1 cells. A**) Mean signal traces (average from 6 wells) from c-NKCC2 expressing LLC-PK1 in the absence (CTR) or in the presence of 50 μM Furosemide during the assay (Furo) were shown. The Tl^+^ influx was inhibited in the presence of Fuorsemide. **B**) The initial rate of Tl^+^ influx deduced from the assay shown in A was reported. The Tl^+^ flux rate was inhibited by about 3 fold in the presence of 50 μM Furosemide. Values are means ± SE of 3 independent experiments. Student’s t test for unpaired data, *p < 0.0001.

As shown in Figure 4A, in c-NKCC2-transfected cells, 30 min of Furosemide preincubation (50 μM) (Furo) clearly inhibited the Cl^-^-dependent Tl^+^ influx compared to control untreated cells (CTR). Calculation of the initial rate of fluorescence increase showed that Tl^+^ influx was inhibited by Furosemide treatment by about 3 fold compared to control conditions (Figure 4B).

Interestingly though, the Tl^+^ influx was not completely abolished even in the presence of higher Furosemide concentrations (data not shown) and it was comparable to that detected in mock-transfected LLC-PK1 cells, likely suggesting the presence of an endogenous furosemide insensitive Cl^-^-dependent Tl^+^ influx in LLC-PK1 cells.

**Figure 5:**
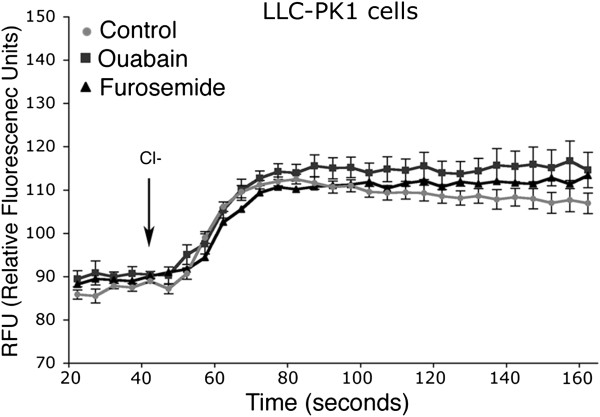
**Tl^+^ influx assay in untransfected LLC-PK1 cells.** Mean signal traces (average from 6 wells) from untransfected LLC-PK1 in the absence (control) or in the presence of 50 μM Furosemide (Furosemide) or 100 μM Ouabain (Ouabain) were shown. No statistically significant differences were observed in the Tl^+^ influx rate in all experimental conditions.

To further verify this hypothesis, furosemide was tested on mock-transfected LLC-PK1 cells. As shown in Figure 5 the weak increase in fluorescence was observed after Cl^-^ addition in mock-transfected LLC-PK1 cells, regardless of whether they were only preincubated with or continuously exposed to furosemide, suggesting the endogenous expression of a Furosemide-insensitive Tl^+^ transport in LLC-PK1 cells (Figure 5, Furosemide).

Although the Tl^+^ flux observed in these cells was Cl^-^-dependent, we definitively excluded that it was mediated by the Na-K-ATPase since it was not abolished by the presence of Ouabain during the assay (Figure 5, Ouabain).

### External Cl^-^ dependency of the c-NKCC2-mediated Tl^+^ transport

**Figure 6:**
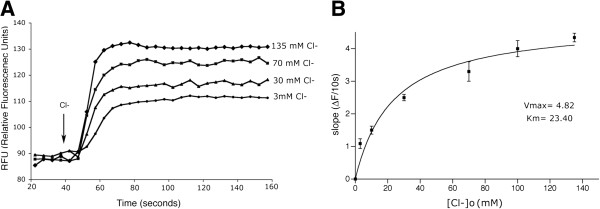
**Dependency of c-NKCC2-mediated Tl^+^ influx on external Cl^-^ in c-NKCC2-expressing LLC-PK1 cells. A**) Tl^+^ influx assays were conducted in assay buffer with varying concentrations of Cl^-^, in which Cl^-^ was replaced by gluconate. Representative raw signal traces after 3, 30, 70, 135 mM Cl^-^ addition were reported. **B**) The initial rate of Tl^+^ influx at the different Cl^-^ concentrations was calculated and plotted against 3, 10, 30, 70, 100, 135 mM [Cl^-^]_o_. Values are means ± SE of 3 independent experiments and the curve represents the best fit of data to a model of activation at a single site. Michaelis-Menten constant Km was deduced.

To assess the dependency of the c-NKCC2-mediated Tl^+^ transport on external Cl^-^ concentration, buffers with different final concentrations (0–135 mM) of Cl^-^, and in which NaCl was replaced by Na-gluconate to maintain appropriate osmolarity, were administered during the assay after Tl^+^ addition. As expected by the expression of NKCC cotransporter, the initial rate of Tl^+^ transport increased proportionately with increasing external concentration of Cl^-^ (Figure 6A). The initial rate of Tl^+^ influx at each Cl^-^ concentration was calculated and plotted against 3, 10, 30, 70, 100, 135 mM extracellular Cl^-^ concentrations. The obtained values were fitted by the model of activation at a single site. A calculation of the affinity of c-NKCC2 for external Cl^-^ revealed a Michaelis-Menten Constant Km at 23.4 (*n* = 8) (Figure 6B).

## Discussion

Technological advances in the past few years have indicated the fluorescence-based methods as among the most useful detection strategies for high throughput screening of drugs. The main advantages associated with this approach are temporal resolution, high sensitivity, simplification of the experimental procedures and miniaturization of the assay and time optimization. In addition, screening assays that are based on optical readout including fluorescence are commonly more robust, suitable for automation, and less expensive than those utilizing radioisotopes or electrophysiological techniques.

A fluorescence-based Tl^+^ influx assay has been recently developed to rapidly measure the activity and to screen for positive modulators of a member of the *SLC* superfamily, KCC2, in mammalian cells, suggesting the feasibility of the Tl^+^ influx through the K-cotransporters as measure of K^+^ fluxes [19].

In the present work we described a fluorescent-based Tl^+^ transport assay that measured the activity of the NKCC2 chimeric construct c-NKCC2, stably expressed at the apical membrane of renal epithelial cells. Although the assay is based on an existing Tl^+^-flux based kit, it has been for the first time adapted to measure the activity of NKCC cotransporters. The assay has been designed in a high-throughput format and has high temporal resolution. The feasibility of the essay was granted by the fact that NKCC cotransporters should be able to transport Tl^+^ ions instead K^+^ and that fluorescent Tl^+^ flux indicators are commercially available. In this assay we used the FluxOR™ Tl^+^ sensitive dye, which is roughly 10-fold more Tl^+^ sensitive, requiring much lower Tl^+^ concentrations for a larger signal window compared to other Tl^+^ dyes [20]. Importantly, this means that the assay can be carried out in physiological saline with normal chloride concentrations. This was an important limitation of other Tl^+^ sensitive dyes, for which chloride-free conditions were necessitated by the solubility coefficient of Thallium chloride, which is about 4 mM at physiological pH.

The activity of NKCC cotransporters is strongly dependent on the phosphorylation of a group of threonine residues in a regulatory domain in its N-terminus [13,23]. One potent stimulus promoting phosphorylation is a fall in cell chloride concentration that causes phosphorylation and activation of SPAK and OSR1, kinases that phosphorylate threonine residues in NKCC regulatory domain [24,25].

Upon the transporter activation, obtained in chloride-free buffers, we have been able to measure a robust Cl^-^-dependent Tl^+^ influx in LLC-PK1 cells stably transfected with the NKCC2 chimeric construct, c-NKCC2.

A number of evidences led us to believe that the Tl^+^ influx measured in our experimental conditions is actually mediated by c-NKCC2. First, substantial Tl^+^ influx was observed exclusively in c-NKCC2-expressing LLC-PK1 cells but not in mock-transfected cells. Second, the Tl^+^ influx exhibited dependency on external Cl^-^ concentrations, a common hallmark of NKCC cotransporters and SLCT12 transporter family in general. Third, the Tl^+^ influx was inhibited by the NKCC inhibitor, Furosemide. Based on these strong indications, we can safely conclude the Tl^+^ influx observed in our assay is due to c-NKCC2 activity.

Interestingly, we observed a residual Tl^+^ influx in LLC-PK1 cells in the presence of either NKCC or Na-K-ATPase inhibitors Furosemide and Ouabain respectively.

Accordingly, an ouabain- and bumetanide- insensitive ^86^Rb^+^ influx has been previously observed in LLC-PK1 cells, suggesting the presence of unknown K^+^ uptake pathways different from NKCC and Na-K-ATPase in these cells [26].

It is possible that LLC-PK1 cells express an endogenous KCC-like activity, although there are no indications for a constitutive expression of KCC transporters in LLC-PK1 cells, and this activity would contribute to the Tl^+^ influx signal. The effect of KCC inhibitors should be tested to definitively address the issue of whether KCC is actually involved in the residual Tl + influx observed in LLC-PK1 cells. However this issue is beyond the scope of this work.

Using different Cl^-^ concentrations in the assay buffer we have been able to measure the affinity of the chimeric c-NKCC2 protein for the external Cl^-^ concentration ([Cl^-^]_o_) (Km = 23.4 mM), which is comparable to that measured for NKCC1 using the ^86^Rb^+^ flux assay [27], found to be 31 mM, and different from that of the NKCC2 counterpart, found to be 12 mM [9].

This value was predictable since this chimera was generated exchanging the C-terminal apical sorting domain of the B mammalian isoform of NKCC2 into the NKCC1 backbone [6]. Indeed, the transmembrane domain of the chimeric construct, responsible for ions transport, belongs entirely to the NKCC1 isoform, thus accounting for the affinity value for Cl^-^ comparable to that measured for NKCC1 by ^86^Rb^+^ influx assay, [27]. One of the critical features of such an assay is its ability to reproduce the results obtained with previously characterized methods.

The obtained Km value for c-NKCC2 showed that the NKCC activity measured by this Tl^+^ influx assay shows comparable kinetic coefficients to those determined with ^86^Rb^+^ influx assay, thus farther validating the method.

Of note, the kinetic behaviour of c-NKCC2-mediated Tl^+^ influx in dependence of [Cl^-^]_o_, did not follow the cooperative kinetics of the two Cl^-^ binding sites as predicted by different functional assays [5,8,27]. However, the issue of whether the NKCC cotransporters family binds ions in a preferred order or displays cooperativity of binding is still far from resolved.

Several studies failed to demonstrate that the two Cl^-^ binding sites behave cooperatively suggesting that the kinetic properties of NKCC-mediated Cl^-^ influx depends on the type of NKCC isoforms, species, cellular models and experimental approaches used in the assays (for details see review [28]). In agreement, Brumback et al. showed that in pyramidal neurons, NKCC1 transport activity in dependence of [Cl^-^]_o_ fits the Michaelis-Menten kinetic model [29].

Indeed, the thermodynamics of Tl^+^ influx in dependence of [Cl^-^]_o_ showed in this study could due to the specific chimeric construct, the cell type and the experimental approach based on fluorescent time courses rather than an end-point measurement of a radioactive compound influx.

We setup this method using polarized epithelial LLC-PK1 cells grown on 96 wells plates and expressing a chimeric NKCC2 at the apical membrane thus reproducing in vitro NKCC2-renal expressing polarized cells.

Although both NKCC1 and NKCC2 are sensitive to loop–diuretics, the renal isoform NKCC2 is the main physiological target of these drugs in the treatment of hypertension. Indeed, the expression of NKCC2 in renal epithelial cells better recapitulates the physiological context where to study the regulation and the pharmacological modulation of this cotransporter.

In other systems, however, the bumetanide-sensitive Tl^+^ uptake has been measured in primary neurons from dorsal root ganglions, likely mediated by the neuronal NKCC1 isoform [30] suggesting the feasibility of this Tl^+^ assay also for the analysis of the NKCC1 isoform.

Moreover, in line of principle, this method could also be applicable to non-polarized mammalian cells such as HEK293, transfected with any NKCC cotransporter constructs. In fact, HEK293 cells have been successfully used in the K^+^ channel functional assay using Tl^+^-sensitive dye in the Flex station device [18] and efficiently transfected with different NKCC constructs displaying the expression of resulting proteins on the overall plasma membrane [5].

It has to be underlined that the Tl^+^ influx assay offers several significant advantages over the other functional assays measuring NKCC cotransporter activity. Compared to radioactive and non radioactive Rb^+^ flux assays, the Tl^+^ influx assay offers high temporal resolution with measurement of real-time changes in ion flux profile within the initial seconds, when ion transport is occurring at a linear rate, rather than minutes, as in the Rb^+^ flux assays in which the end point Rb^+^ intracellular accumulation is measured. Importantly, as a continuous assay, the Tl^+^ flux assay is easier to perform and inherently offers improved sensitivity and accuracy over endpoint assay. Compared to a NH4^+^ uptake assay measured with a pH-sensitive fluorescent dye, the Tl^+^ influx assay is direct functional assay with better resolution and sensitivity.

## Conclusions

The new functional assay we described here would be extremely useful in the high-throughput screening and characterization of new NKCC inhibitors as anti-hypertensive drugs. Moreover, the chimeric NKCC1-NKCC2 construct used in this work, functionally expressed at the apical membrane of polarized LLC-PK1 cells, will allow us to explore factors that regulates NKCC2 sorting and function in a more physiological context such as a renal epithelial polarized cell system.

## Methods

### Reagents

The FluxOR™ Thallium detection kit was from Life Technologies™ (http://www.lifetechnologies.com). All chemicals were purchased from Sigma-Aldrich® (http://www.sigmaaldrich.com).

### Cell culture

c-NKCC2-LLC-PK1 cells were generated as previously described [6] and cultured in DMEM (Life Technologies™) supplemented with 10% fetal bovine serum (Life Technologies™) and 1% penicillin-streptomycin (Life Technologies™) in a humidified 5% CO_2_, 95% O_2_ incubator at 37°C.

### Immunofluorescence

c-NKCC2-LLC-PK1 cells were grown on glass coverslips and subjected to immunofluorescence when they reached the confluency. Cells were fixed in cold methanol for 10 min. After three washes in phosphate-buffered saline (PBS), cells were blocked in saturation buffer (1% bovine serum albumine in PBS) for 20 min at room temperature (RT) and incubated with the primary antibodies for 2 h at RT in saturation buffer. After 3 washes in PBS cells were incubated with the appropriate Alexafluor conjugated secondary antibodies (Life Technologies™) for 1 h at RT. Primary antibodies used were: polyclonal anti-HA antibody (Covance, 1:1000) to detect c-NKCC2, the monoclonal anti-α subunit of Na^+^/K^+^ATPase antibody for the detection of the endogenous Na-K-ATPase (Sigma, 1:500) and the polyclonal anti-NKCC2 antibody (Millipore, 1:500) for the detection of endogenous NKCC2 in both LLC-PK1 cells and rat kidney slides. Confocal images were obtained with a laser scanning fluorescence microscope Leica TSC-SP2 (HCX PL APO, ×63/1.32–0.60 oil objective); 8-bit images were saved at 1024 × 256 and acquired using the Leica Confocal Software®.

### Fluorescence acquisition

Fluorescence signal was acquired using the FlexStation II devise from Molecular Devices (http://www.moleculardevices.com/), MDS Analytical Technologies, USA, equipped to perform functional cellular assays and to analyze real time fluorescence kinetic data in the 96-well format. The instrument consists of an incubated cabinet with fluorometer and integrated 96 channel pipettor which is able to transfer compounds from one compound microplate to the assay plate, allowing rapid kinetic assays. Data acquisition was performed by SoftMax Pro software.

### Tl^+^ influx assay by the FlexStation device

Cells were seeded in 96-well, black-walled, clear-bottomed (Corning, http://www.corning.com) at a density of 5 × 10^4^ cells per well 48 h before the assay at which time cells were 100% confluent.

On the day of the assay, the cell culture medium was replaced with 100 μl/well of the FluxOR™ chloride-free buffer (Component E) containing the Tl^+^-sensitive fluorogenic indicator dye, FluxOR™ reagent (Component A) and Probenecid (Component D) according to the manufacturer’s instructions. The FluxOR™ reagent is a non fluorescent indicator dye, which is loaded into cells as a membrane-permeable acetoxymethyl (AM)-ester. Once inside the cell, the non-fluorescent AM ester form of the FluxOR™ dye is cleaved by endogenous esterases into a fluorogenic Tl^+^-sensitive indicator. The Tl^+^-sensitive form is retained within the cytosol and its extrusion is inhibited by water-soluble Probenecid, which blocks organic anion pumps.

Dye loading was allowed to proceed for 1 h at 37°C, then cells were washed twice in 100 μl/well assay buffer (in mM: 135 Na-gluconate, 1 mM MgCl_2_, 1 mM Na_2_SO4, 1 mM CaCl_2_, 15 mM Na-HEPES, pH 7,4). The wash buffer was discarded, cells were left in 100 μl of assay buffer and loaded onto the FlexStation device. When required, 50 μM furosemide was added in all these steps.

Ions to be transported by NKCC2 (Cl^-^ and Tl^+^) were prepared in the compound assay plate as 6× concentrated solutions in the assay buffer to give a final concentration of 135 mM NaCl and 2 mM Tl_2_SO_4_ after addition in the assay plate.

FluxOR dye was excited at 490 nm and detected at 520 nm using dual monochromators. Time course fluorescence data were recorded over a 80 s period. We assayed each well for Cl^-^-dependent Tl^+^ transport by continuously recording fluorescence for 15 s (baseline), then for 15 s after rapid automated addition of 20 μL of 6× Tl_2_SO_4_ and for 40 s after automated addition of 20 μL of 6× NaCl. To optimize the number of data points, we set the instrument to read out sequentially the top half of the plate and the bottom one. Each time point was, thus, obtained every 2 s. The initial rate of Tl^+^ transport was deduced by analyzing the linear increase of fluorescent signals within the initial 15 s following NaCl addition.

### Data analysis

Relative initial rates of Tl^+^ transport were calculated as the slope of the linear kinetic phase of fluorescent signal within the first 20 s after Cl- addition.

Data were collected from at least 6 trials for each concentration and fit with a single-site binding curve using Graphpad prism software (Graphpad software). Michaelis-Menten constant Km was deduced.

## Authors’ contributions

MC designed the experiments, developed the experimental work, performed data analyzes and wrote the paper. FR conducted the Tl + influx assays. ST provided a technical support. GP conducted microscopy confocal analysis. MS supervised the study, and supervised the writing of the article. All authors read and approved the final manuscript.

## Authors’ information

The corresponding author Carmosino M, is currently an Associate Professor in Physiology at the University of Basilicata, Italy. She has been a Faculty Member of the Department of Cellular and Molecular Physiology at the Yale University School of Medicine from 2006 to 2010, where she made important observations on the polarized trafficking and ion transport kinetics of the renal cotransporter NKCC2 (*Carmosino et al*. Mol Biol Cell. 2008 Oct; 19(10):4341–51; Pedersen M, *Carmosino M*, Forbush B., J Biol Chem. 2008 Feb 1;283(5):2663–74; *Carmosino et al*. Mol Biol Cell. 2010 Nov 15; 21(22): 3985–97). She collaborated with Professor Biff Forbush and Professor Mike Caplan, who made profound contributions on the discovery and characterization of the NKCC cotransporters and developed automatized devices to measure ion fluxes mediated by this cotransporter family in oocytes and mammalian cells.
